# Early detection of large‐vessel occlusion stroke after cardiac surgery using CT angiography leading to early recanalization with endovascular thrombectomy

**DOI:** 10.1002/ccr3.4246

**Published:** 2021-05-19

**Authors:** Kiyoshi Takemoto, Masaaki Sakuraya, Michitaka Nakamura, Hidetsugu Maekawa, Kazuo Yamanaka, Kazuaki Atagi

**Affiliations:** ^1^ Division of Critical Care Medicine Nara Prefecture General Medical Center Nara Japan; ^2^ Department of Emergency and Intensive Care Medicine JA Hiroshima General Hospital Hiroshima Japan; ^3^ Department of Neurosurgery Nara Prefecture General Medical Center Nara Japan; ^4^ Department of Cardiovascular Surgery Nara Prefecture General Medical Center Nara Japan

**Keywords:** endovascular thrombectomy, large‐vessel occlusion stroke, open‐heart surgery, three‐dimensional computed tomography angiography

## Abstract

CT angiography may be useful for early diagnosis of ischemic stroke after cardiac surgery. When patients diagnosed with large‐vessel occlusion, endovascular thrombectomy may be a therapeutic option and may improve their neurological complications.

## Graphical abstract

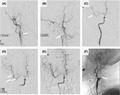

## 1 INTRODUCTION

Acute ischemic stroke after cardiac surgery is one of the most devastating complications. This case report suggests the efficacy of CT angiography for early diagnosis of ischemic stroke after cardiac surgery. This patient was diagnosed with large‐vessel occlusion and performed successful endovascular thrombectomy without neurological complications.

Acute ischemic stroke is one of the most devastating complications that can occur after cardiac surgery. The incidence of ischemic stroke after a coronary artery bypass graft (CABG) was reported to be 0.9%–2.7%,^1^, ^2^ with 40% occurring during surgery and 58% occurring after surgery.^3^ In recent years, although the frequency of perioperative stroke after CABG has been improved with preoperative brain and carotid artery examinations, the aortic nontouch technique, and off‐pump CABG (OPCAB),^4^ postoperative ischemic stroke is still a severe complication.

Magnetic resonance imaging (MRI) is known to be useful for the early diagnosis of acute ischemic stroke. However, it is difficult for patients who have just received open‐heart surgery to have MRI because several associated devices, such as pacing wires, Swan‐Ganz catheters, drainage tubes, and intubation with mechanical ventilation, make MRIs almost impossible to perform. This case report suggests the efficacy of three‐dimensional computed tomography angiography (3D‐CTA) of the brain for the early diagnosis of acute ischemic stroke, due to the large‐vessel occlusions that often occur in this setting.

On the other hand, recanalization with endovascular thrombectomy for acute ischemic stroke has proven to be effective as a treatment,^5^, ^6^ but there are limited reports of cases treated with endovascular thrombectomy after open‐heart surgery because patients with major surgery were excluded from these trials. Nonetheless, some successful cases of endovascular thrombectomy for acute ischemic stroke after cardiac surgery have been reported.^7^, ^8^ We report an instance of recanalization with endovascular thrombectomy without any complications.

## 2 CASE PRESENTATION

**FIGURE 1:**
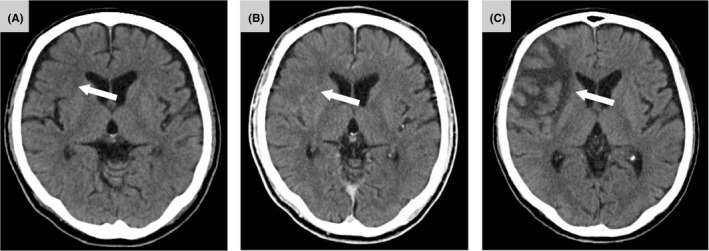
Initial CT of the brain indicated slightly low density of the right frontal and insular cortex (A, arrow). Followed‐up CT of the brain after recanalization showed no intracerebral hemorrhage, and low density of the right frontal and insular cortex were a little more apparent (B, arrow). Followed‐up CT of the brain at day 7 revealed ischemic edema of the frontal operculum and temporal pole (C, arrow)

**FIGURE 2:**
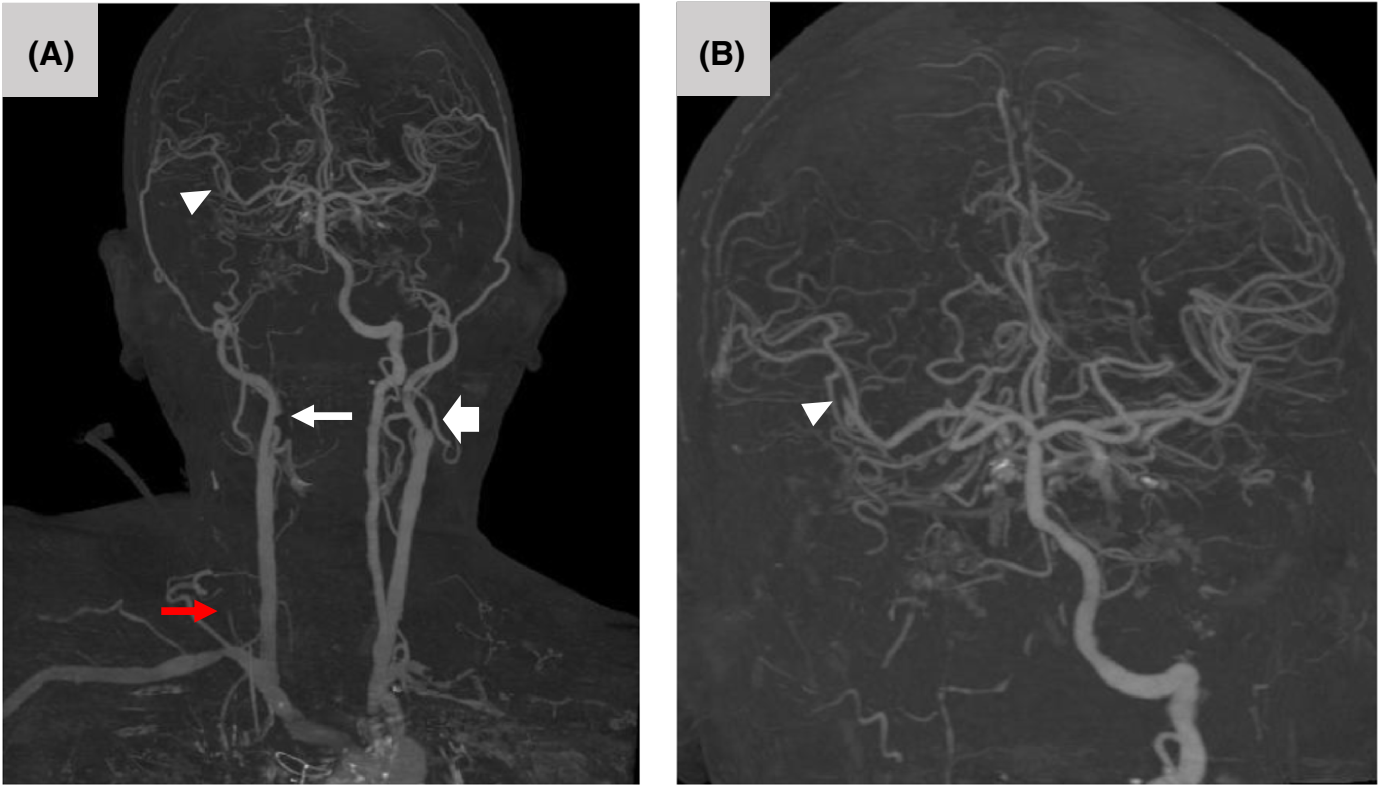
Three‐dimensional reconstructed images of CT angiography of the neck and brain showed occlusion of the origin of the right ICA (A, white arrow), the origin of the left ICA (A, wide arrow), the origin of the right vertebral artery (A, red arrow), and the M2 segment of the middle cerebral artery (A, B, arrowhead)

**FIGURE 3:**
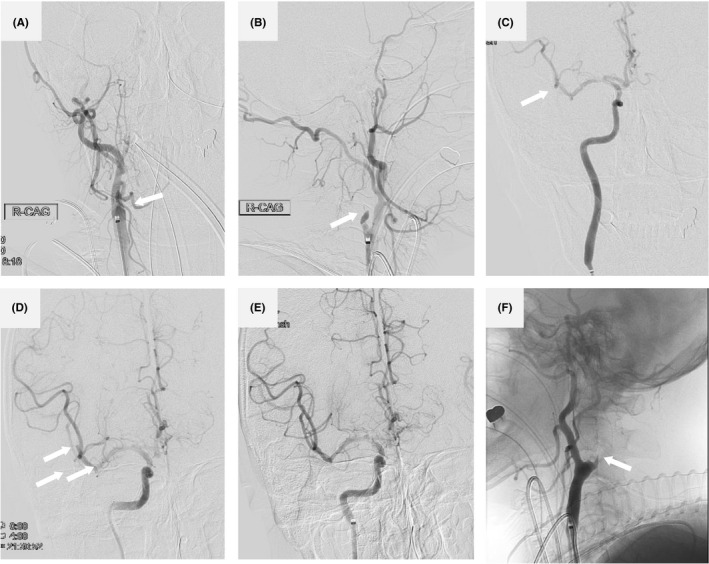
Cerebral angiography and endovascular thrombectomy findings. An initial injection of the right common carotid artery indicated occlusion of the right cervical ICA (A, B, arrow, A, AP view, B, lateral view). Manual aspiration with a syringe was performed with the balloon inflated to arrest the flow of the right ICA. Sizable clots were removed. An injection of the right ICA showed completely recanalized ICA (C, arrow, AP view), with moderate stenosis at the origin and occluded M2 segment of MCA (C, D, arrow, AP view). Endovascular thrombectomy using a Solitaire (Medtronic) stent retriever was performed for occlusion of the MCA, and a subsequent ICA injection demonstrated excellent reperfusion of the MCA with a modified thrombolysis in cerebral infarction score of 2A (E, arrow, AP view). Although the occlusion of the left ICA was detected (F, arrow, lateral view), recanalization was not performed because it was not the responsible vessel of neurological deficits

A 73‐year‐old Japanese man was admitted for OPCAB due to symptomatic angina. His medical history included hypertension, chronic kidney disease, and asymptomatic bilateral internal carotid artery (ICA) stenosis. Carotid ultrasonography showed 94.0% of stenosis in the right ICA area with a peak velocity of 283.2 cm/s. The left ICA showed a peak velocity of 595.2 cm/s, although the stenosis rate of area could not be counted. Coronary angiogram showed 99% stenosis of the left anterior descending coronary artery (LAD), 99% stenosis of the distal segment in the right coronary artery (RCA), and 90% stenosis in the proximal segment of the left circumflex coronary artery (LCX). Left ventricular angiography showed severe hypokinesis of the posterior wall with an ejection fraction of 40%. Although carotid ultrasonography demonstrated bilateral ICA stenosis, preoperative CT of the brain showed no ischemic stroke with any neurological symptoms. On the basis of these findings, there was no indication for preceding carotid endarterectomy or carotid artery stenting, and revascularization was selected performing OPCAB to avoid perioperative stroke. OPCAB was performed, using the left internal thoracic artery to graft the LAD, the saphenous vein graft (SVG) for the posterior descending artery of the RCA, and the SVG for the posterior lateral of the LCX. The anesthesia and operation times were 6 h 11 min and 4 h 11 min, respectively. He was hemodynamically stable during and after surgery. Four hours after the operation in intensive care unit (ICU), he regained consciousness from the anesthesia, demonstrating left‐sided hemiparesis with sensory disorder. We suspected a perioperative stroke because of his bilateral ICA stenosis. His National Institutes of Health Stroke Score (NIHSS) was 10 out of 42 points at that time. A CT of the brain indicated a slightly low density of the right frontal and insular cortex (Figure 1A). Additional CT with a contrast agent as 3D‐CTA of the neck and brain was performed and exhibited multiple large‐vessel occlusions: the origin of the bilateral ICA, the origin of the right vertebral artery, and the M2 segment of right middle cerebral artery (MCA; Figure 2A,B). We believed that there was a small ischemic core and that the brain tissue would be largely salvageable by recanalization of the right ICA and MCA; hence, endovascular thrombectomy was suggested. An initial injection of the right common carotid artery indicated occlusion of the right cervical ICA (Figure 3A,B). Manual aspiration with a syringe was performed with the balloon inflated to arrest the flow of the right ICA. Sizable clots were removed. An injection of the right ICA showed completely recanalized ICA (Figure 3C), with moderate stenosis at the origin and occluded M2 segment of MCA (Figure 3C,D). Endovascular thrombectomy using a Solitaire (Medtronic) stent retriever was performed for occlusion of the MCA, and a subsequent ICA injection demonstrated excellent reperfusion of the MCA with a modified thrombolysis in cerebral infarction score of 2A (Figure 3E). Although the occlusion of the left ICA was detected (Figure 3F), it was not the responsible vessel of these neurological deficits. The recanalization was not performed because there was a high risk of distal embolism, which might have caused new deficits, or bleeding due to duration of angiography after open‐heart surgery. Since recanalization of the responsible vessel was most important first of all, the routine four vessel angiograms were not performed. After recanalization, followed‐up CT of the brain showed no intracerebral hemorrhage and slightly low density of the right frontal and insular cortex (Figure 1B). His left‐sided hemiparesis improved immediately after the thrombectomy. We administered 100 mg of aspirin daily for this acute ischemic stroke and ICA stenosis. Followed‐up CT of the brain at day 7 revealed ischemic edema of the frontal operculum and temporal pole (Figure 1C). His neurological examination found improvement gradually, and he left the ICU at day 9. He continued rehabilitation for his residual neurological deficits. Finally, his left‐sided hemiparesis persisted only slightly, and the NIHSS was improved two out of 42 points. The patient was discharged from the hospital on day 46. He has been followed up as an outpatient. Subsequently, carotid artery stenting was performed for his remaining right ICA stenosis 6 months later. He exhibited no recurrence of symptomatic ischemic stroke or angina for 1 year with his medical treatment.

## 3 DISCUSSION

There are various mechanisms of perioperative ischemic stroke. A previous study has suggested that the cause of ischemic stroke is associated with blocked and released ascending aorta from cardiopulmonary bypass, ascending aortic procedure, thromboembolism, atheromatous thrombus, air embolism, and hypoperfusion.^9^ Although his past medical history indicated the risk of ischemic stroke due to ICA stenosis, we could not detect the cause of thrombus during or after the operation. Based on his severe carotid stenosis, a hemodynamic infarct may have been considered in the initial neurological assessment. It is important to note that it might be difficult to evaluate neurological findings and detect the onset time of stroke accurately under the influence of anesthetics after surgery. It has been suggested and advised against treating relevant carotid stenosis prior to cardiac surgery.^10^, ^11^ The decision for carotid angioplasty or endarterectomy not to treat the occluded carotid artery may depend upon patency of the Circle of Willis. In this case, carotid angioplasty or endarterectomy should have been performed prior to CABG to avoid critical neurological complications.

A recombinant tissue plasminogen activator is an established treatment for acute ischemic stroke; however, major surgery, such as open‐heart surgery, is contraindicated owing to the high risk of bleeding. Nevertheless, the efficacy and safety of endovascular thrombectomy are still relatively unexplored, and it may be an important therapeutic option in these cases. If we suspect a new onset of stroke, a CT of the brain is the most important imaging examination. It may be difficult to diagnose early‐onset ischemic stroke in cases with no obscuration of the lentiform nucleus, loss of insular ribbon, loss of gray‐white differentiation, or hyperdense MCA sign. In this case, the CT of the brain indicated a slightly low density of the right frontal and insular cortex (Figure 1A), but it was difficult to confirm acute ischemic stroke and detect occlusion of the responsible artery. On the basis of these considerations, 3D‐CTA with a CT of the brain may be a useful imaging examination for early confirmation of whether to perform endovascular thrombectomy or not.

In this case, the thrombus of the right ICA and the M2 branch of the MCA was removed, and recanalization with endovascular thrombectomy was successful, with no complications. If endovascular thrombectomy is to be considered as one of the therapeutic options in these situations, it is of utmost importance to carry out neurological examinations and angiography to detect occlusion of the responsible artery. We must consider hemodynamic status, laboratory analysis, and any other condition experienced by the patient.

## 4 CONCLUSION

This study presents the case of a symptomatic Japanese patient diagnosed with acute ischemic stroke due to large‐vessel occlusion after open‐heart surgery using 3D‐CTA. It suggests that endovascular thrombectomy may be a therapeutic option in such cases.

## CONFLICT OF INTEREST

None declared.

## AUTHOR CONTRIBUTIONS

KT: has made substantial contributions to conception and interpretation of data, and wrote the manuscript; MS, MN, HM, KY, and KA: cared for the patient and made contributions to the paper. All authors: reviewed and contributed to the present form of the manuscript.

## ETHICAL APPROVAL

The patient gave written consent to report his case and the imaging. The patient gave written consent to report his case and the imaging.
